# Effect of Nitazoxanide and Probiotic Treatment on Bangladeshi Children with Cryptosporidiosis

**DOI:** 10.4269/ajtmh.23-0914

**Published:** 2025-02-11

**Authors:** Zannatun Noor, Biplob Hossain, Nishad Tasnim Mithila, Amena Khatun, Sultan Mahmud, Aleya Ferdous, Bipasha Akhter, Masud Alam, Carol A. Gilchrist, Rashidul Haque, William A. Petri

**Affiliations:** ^1^ Infectious Diseases Division, International Centre for Diarrheal Disease Research, Bangladesh, Dhaka, Bangladesh;; ^2^ Nutrition Research Division, International Centre for Diarrheal Disease Research, Bangladesh, Dhaka, Bangladesh;; ^3^ North South University Genome Research Institute, Dhaka, Bangladesh;; ^4^ Department of Molecular Genetics and Microbiology, Vaccine Testing Center, University of Vermont, Burlington, Vermont;; ^5^ Department of Medicine, University of Virginia, Charlottesville, Virginia

## Abstract

*Cryptosporidium* spp. is a cause of diarrhea morbidity and mortality in children under 5 years of age. In addition, asymptomatic infections can have a negative impact on growth and development. In low- and middle-income countries where a greater number of infants may be malnourished, the results of treating cryptosporidiosis with the only Food and Drug Administration-approved drug nitazoxanide (NTZ) have been inconsistent. Malnutrition is both a risk factor for cryptosporidiosis and a consequence of infection with this parasite. Treatment with the probiotic *Lactobacillus reuteri* DSM 17938 has been shown to assist in nutritional recovery and the restoration of gut health. In this pilot randomized clinical trial, we examined whether combined probiotic and NTZ treatment could result in the reduction in parasitemia and infection-associated growth stunting in undernourished children. *Cryptosporidium* spp.-positive Bangladeshi children with a weight-for-length *Z* score between −1 and −3 were randomly assigned to one of three groups. Group 1 (*n* = 26) received NTZ and *Lactobacillus*, group 2 (*n* = 28) received NTZ along with a placebo, and the third control group (*n* = 10) received standard care. There was no difference in the duration of infection or improvement in child anthropometric measurements in any treatment group compared with control. Therefore, this pilot study does not provide support for treatment with NTZ, *Lactobacillus*, or the two in combination as an effective means of reducing the duration of *Cryptosporidium* spp. infection or improving growth in growth-stunted children.

## INTRODUCTION

Human cryptosporidiosis is a diarrheal illness resulting from infection with apicomplexan protozoan parasites belonging to the genus *Cryptosporidium* spp.^1^ In immunocompetent individuals, infection with *Cryptosporidium* spp. typically leads to either subclinical asymptomatic disease or self-limiting moderate to severe watery diarrhea that naturally resolves within 2–3 days of infection.^2^^,^^3^ Both subclinical and diarrheal *Cryptosporidium* spp. infections can also have long-term impacts on child growth and development that exacerbate the effects of undernutrition in food-insecure households.^4^^,^^5^ In disadvantaged communities in South America, sub-Saharan Africa, and Asia, the prevalence of cryptosporidiosis (subclinical or symptomatic) was 65% (the Malnutrition and Enteric Disease study).^6^ It was also among the most frequent enteric pathogens detected in the Global Enteric Multicenter Study (GEMS), which focused on the impact of enteric diseases on children’s health in sub-Saharan Africa and South Asia. In GEMS, *Cryptosporidium* spp. was the second-most common pathogen correlated with moderate-to-severe diarrhea in children below the age of 2 years and emerged as the predominant pathogen connected to mortality in infants of ages between 12 and 23 months.^7^ In children, both exclusive breastfeeding and higher levels of anti-*Cryptosporidium* IgA in breast milk have been found to be protective.

Clinical treatment of symptomatic cryptosporidiosis is usually limited to oral rehydration and other supportive care.^8^^,^^9^ The only antiprotozoal drug approved by the U.S. Food and Drug Administration (FDA) to treat cryptosporidiosis in children (older than 1 year of age) and adults is nitazoxanide (NTZ).^10^^,^^11^ However, NTZ has been observed to be ineffective when given to those with compromised immune systems.^12^ In clinical trials involving 25 HIV-negative but malnourished Zambian children, its effectiveness rates were 56% for diarrhea resolution and 52% for oocyst clearance compared with 23% and 14%, respectively, in 25 controls.^13^^,^^14^ In 28 Egyptian children, 7 days of NTZ treatment, however, resulted in oocyst clearance in 80% of patients compared with the natural clearance rate of 41% in 27 controls.^15^

Probiotic interventions can modify systemic and mucosal innate and acquired immunity. It is hypothesized that the host microbiome can protect against *Cryptosporidium* spp. colonization.^16^ Both *Lactobacillus acidophilus* and *Lactobacillus reuteri* have been observed to shorten the duration and quantity of *Cryptosporidium parvum* oocysts shed in the feces of experimentally infected mice.^17^^,^^18^ Probiotic treatment with *L. reuteri* has been reported to induce intestinal epithelial proliferation, aid in the repair of epithelial damage, and subsequently, reduce inflammation.^19^ The probiotic strain DSM 17938 of *L. reuteri* has demonstrated both acceptability and safety when administered to infants in Bangladesh.^20^ In this study, we tested whether cotreatment of NTZ and *Lactobacillus* could reduce infection duration, host stunting, and oocyst burden in stool, which would potentially reduce parasite transmission.

## MATERIALS AND METHODS

### Study site and population.

This study was carried out among Bangladeshi children between 12 and 36 months old living in the Mirpur slums. This study was a pilot, open-blind, three-arm, randomized, controlled clinical trial comprising an initial baseline screen to identify *Cryptosporidium*-positive children. The *Cryptosporidium*-positive children who met the inclusion criteria of having no history of antibiotic use in the last month and whose weight-for-length *Z* score (WLZ) fell between −1 and −3 were selected for enrollment into the study. After securing informed consent, a standard questionnaire was administered to the parents or guardians of the children to gather demographic information and details of any diarrheal symptoms. For the next 3 weeks, twice weekly home visits were conducted by trained field investigators to monitor the child’s health. Fecal samples collected from both diarrheal and asymptomatic surveillance stools were processed to extract fecal DNA. Diagnostic quantitative polymerase chain reactions (qPCRs) were run to quantify the number of oocysts being shed. The day of enrollment was counted as study day 0; subsequent study visits occurred on days 4, 8, and 20 postenrollment. To evaluate any longer-term treatment outcomes on child growth, home visits to collect anthropometric data were carried out at 90 and 180 days.

### *Cryptosporidium* spp. QUIK CHEK rapid antigen assay.

Stool samples were collected at home by study personnel on the day of enrollment (day 0) and on study visits (days 4, 8, and 20). These samples were immediately placed in a cold box before transportation to the field laboratory at the Mirpur study site. *Cryptosporidium* spp. infection was detected by use of the rapid TechLab (Blacksburg, VA) *Cryptosporidium* and *Giardia/*QUIK CHEK immunological test (T30407), which was performed as directed by the manufacturer’s instructions.^21^

### Anthropometric measurements of children.

At the time of enrollment (day 0) as well as during the 90- and 180-day follow-up visits, information on children’s height, weight, and midupper-arm circumference (MUAC) was collected. Height was measured using a precision Shorr Board (Weigh and Measure, LLC, Olney, ML), and the standing weight of the children was determined using a digital scale (HD-314; Tanita, Arlington Heights, IL). The tricolored insertion MUAC tape was used to measure arm circumference at the midpoint between the shoulder and elbow of the extended left arm. WHO’s Anthro software v. 3.2.2 (Geneva, Switzerland) was used to calculate the height-for-age* Z* score (HAZ) or length-for-age *Z* score, the weight-for-age *Z* score (WAZ), and the weight-for-height *Z* score (WHZ) or WLZ.

### Random assignment to sample groups and sample collection.

*Cryptosporidium*-positive children were randomized by using a simple random sampling selection procedure and assigned to one of the three treatment groups (control, NTZ plus placebo, and NTZ plus the probiotic). After the *Cryptosporidium*/*Giardia* QUICK CHEK was used to identify infected children, other remaining stool samples and the stool samples collected subsequently on days 4, 8, and 20 days after enrollment were sent to the icddr,b Parasitology Laboratory in a temperature- monitored cold box. At icddr,b, the remainder of the sample was aliquoted and stored at −70°C.

### Intervention procedures.

The *L. reuteri* DSM 17938 probiotic was acquired from the Swedish healthcare company Bio Gaia (Stockholm, Sweden). The NTZ oral solution (ZOX-30 mL; 20 mg NTZ per 1 mL) and the lactulose placebo (United States Pharmacopeia 3.35 g/5 mL; OsmoLax; Key Pharmaceuticals, North Ryde, NSW, Australia) were purchased at the local pharmacy (Square Pharmaceuticals Ltd., Dhaka, Bangladesh). The standard NTZ treatment FDA guideline for children 1 year of age or older (100 mg/5 mL administered every 12 hours with food) was used in this study. The dosage and duration of treatment with the *L. reuteri* DSM 17938 probiotic were selected based on previous studies of its efficacy and safety, and the dosage consisted of five drops (2 × 10^8^ colony forming unit) of *L. reuteri* DSM 17938 administered every 12 hours with food.^22^ The lactulose placebo (five drops at a concentration of 250 mg/mL) was administered twice daily for 7 days by our study field assistant.

Children were divided at random into the three treatment groups. Group 1 received the NTZ treatment for 3 days and probiotics for 7 days (NTZ plus probiotic group). Group 2 received NTZ treatment for 3 days and placebo for 7 days (NTZ plus placebo group). Group 3 was provided with the standard supportive care for *Cryptosporidium* infections (control group), which involves monitoring for dehydration in diarrheal cases and repletion of electrolyte losses by oral rehydration.^23^ As per standard protocol, after the intervention, a member of the study field staff stayed at the participant’s house for 60 minutes to monitor the child and document any adverse events occurring after the intervention (nausea or vomiting). Patients in this study did not adversely react to any of the administered treatments.

### Multiplex real-time polymerase chain reaction.

Fecal DNA was extracted from the stool samples sent to the icddr,b Parasitology Laboratory using the previously described modified Qiagen stool DNA extraction protocol (QIAamp Fast DNA Stool Mini kit, catalog no. 51604; Sigma-Aldrich, Burlington, MA).^24^ The modifications added were a 95°C incubation step and a bead-beating step to homogenize and suspend the stool sample in InhibitEX buffer. During DNA extraction, Phocine Herpes Virus 1 (PhHV) was added as an internal positive control. To detect possible contamination, one sample of the InhibitEX buffer alone was included as an extraction blank.

The CFX96 Real-Time Detection System (BIO-RAD, Hercules, CA) was used to amplify, detect, and analyze data. A multivalent real-time TaqMan diagnostic qPCR assay was then run on the extracted DNA as previously described.^25^^,^^26^ This assay targeted both the *Cryptosporidium* spp. 16S small-subunit ribosomal RNA gene and the gB gene of the PhHV internal control.^25^^,^^26^ Both negative and positive controls were included in each qPCR plate, and the amplification reaction involved 40 cycles of 15 seconds at 95°C, 30 seconds at 60°C, and 30 seconds at 72°C. Positive samples were defined as those with a quantification cycle value of ≤36.

### *Cryptosporidium* spp. antigen ELISA test.

The *Cryptosporidium* spp. II ELISA kit (TechLab) was used to identify the *Cryptosporidium* spp. antigen in fecal samples using the manufacturer’s instructions. Optical density (OD) values at a single wavelength of 450 nm were used to interpret the assay, with OD values of ≥0.150 considered as positive for *Cryptosporidium*. We tested all of the time points of a child on the same plate to reduce the batch effect. A standard curve was included to confirm that the OD values fell within the linear range of the assay.

## STATISTICAL ANALYSES

For statistical analysis, data were entered into FoxPro v. 2.6 (Microsoft Corp, Redmond, WA), cleaned, and exported into SPSS v. 20 (IBM Corp., Armonk, NY) and GraphPad Prism v. 5 (Dotmatics, Boston, MA). The normality of the data was assessed using the Kolmogorov–Smirnov test. A statistically significant level of ≤0.05 was used with a 95% CI. Group comparisons were carried out using the Fisher exact *t*-test for nominal data to identify significant differences between groups. To compare the results obtained at different time points, either a paired *t*-test or a nonparametric Wilcoxon matched-pairs signed rank test was used. To compare results between groups, a Student’s *t*-test or a Mann–Whitney *U* test was used. Kaplan–Meier survival curves were used to compare infection clearance in the different groups, and the log-rank (Mantel–Cox) test was used to assess statistical significance.

## RESULTS

### Demographic and anthropometric characteristics.

**Figure 1.**
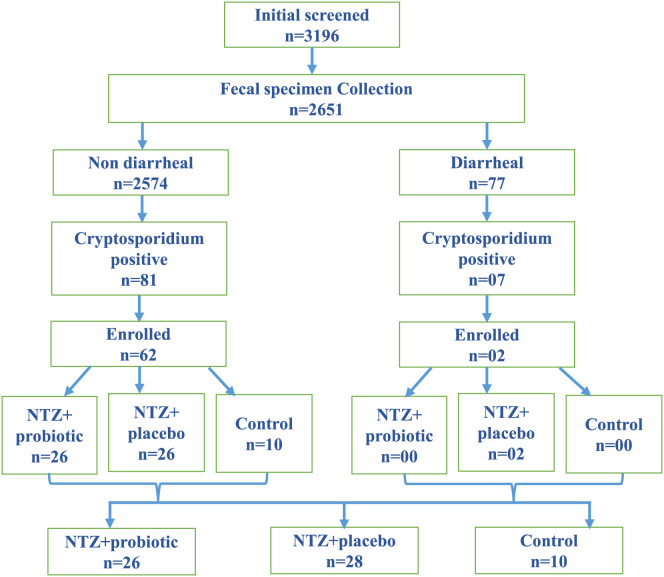
The study design flowchart shows the details of the randomized, controlled trial. NTZ = nitazoxanide.

A total of 3,196 children were initially screened for this study, and 2,651 agreed to provide stool samples at the time of enrollment (Figure 1). The TechLab *Giardia/Cryptosporidium* QUIK CHEK assay identified 88 of these samples to be *Cryptosporidium* spp.-positive (3.3%; *N* = 88/2,651). A standard questionnaire was administered to the parents or guardians of these children to gather information on diarrheal symptoms in the previous fortnight, and the Bristol stool scale was used to characterize the stool specimens; 77 (2.9%) of the samples were classified as being collected from children with diarrhea. Approximately 9.1% of the diarrheal specimens (*n* = 7/77) and 3.1% (*n* = 81/2,574) of the nondiarrheal specimens were *Cryptosporidium* positive. Of the initial 88 QUIK CHEK-positive participants, 64 of the recruited children met our inclusion criteria (no history of prior antibiotic use 3 months before enrolment and WLZ that fell between −1 and −3) and were enrolled in this study. These included only two of the diarrheal cases. The open-blinded random sampling selection procedure used to assign the children to the treatment groups resulted in the two symptomatic cases being assigned into the NTZ plus placebo group (Figure 1). The demographic and anthropometric characteristics of the 64 participants enrolled in the different groups are summarized in Table 1. The study included 26 children in the NTZ plus probiotic treatment arm, 28 children in the NTZ plus placebo treatment arm, and 10 children in the control arm, in which children received the normal standard supportive care provided for *Cryptosporidium* spp. infections (monitoring of child health with oral rehydration as necessary).^23^ Because of the small number of children enrolled in this pilot study, a significant difference arose by chance in the gender of the enrolled children assigned to the NTZ plus probiotic arm, in which only 28.5% of the children were male versus ∼60% in the other study groups (*P* = 0.04)*.* The children assigned to the NTZ plus probiotic, NTZ plus placebo, and control arms did not otherwise significantly differ regarding weight, height, and MUAC or in the derived HAZ, WAZ, or WHZ.

**Table 1:** Demographic and anthropometric characteristics of the participants enrolled in the nitazoxanide plus probiotic arm, the nitazoxanide plus placebo arm, and the control arm

Variables	NTZ + Probiotic (*n* = 26)	NTZ + Placebo (*n* = 28)	Control (*n* = 10)	*P*-Value
Male, *n* (%)	8 (28.5)	18 (64)	6 (60)	0.04
Age, months (±SD)	23.70 (±6.45)	20.81 (±6.06)	23.98 (±4.85)	0.16
Weight, kg (±SD)	9.71 (±1.69)	9.17 (±1.11)	10.24 (±1.21)	0.09
Height, cm (±SD)	81.30 (±6.81)	78.98 (±4.89)	84.08 (±5.39)	0.06
MUAC, cm (±SD)	14.06 (±1.07)	13.97 (±0.87)	14.40 (±1.02)	0.51
HAZ (±SD)	−1.46 (±1.21)	−1.59 (±1.31)	−0.72 (±1.15)	0.17
WAZ (±SD)	−1.54 (±1.01)	−1.75 (±1.01)	−1.28 (±0.70)	0.39
WHZ (±SD)	−1.11(±0.81)	−1.31 (±0.81)	−1.27 (±0.80)	0.67

HAZ = height-for-age *Z* score; MUAC = midupper-arm circumference; NTZ = nitazoxanide; WAZ = weight-for-age *Z* score; WHZ = weight-for-height *Z* score. The *P*-value for the difference was determined by the analysis of variance statistical *t*-test for continuous data and the χ^2^ test for the categorical data. Values are expressed as mean ± SD.

### NTZ treatment with or without probiotic did not significantly reduce the duration of a *Cryptosporidium* spp. infection.

**Figure 2.**
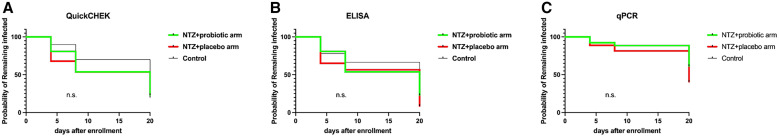
*Cryptosporidium* spp. infection prevalence. This was assessed using (**A**) QUIK CHEK, (**B**) ELISA, and (**C**) quantitative polymerase chain reaction (qPCR) techniques on days 4, 8, and 20 after enrollment. The *y* axes indicate the probability of remaining infected; the *x* axes indicate the days since the initial positive stool sample was collected. Lines represent the probability of remaining infected over time in the different groups: nitazoxanide (NTZ) plus probiotic (green lines), NTZ plus placebo (red lines), and control (black lines). The *P*-value for the difference between groups was determined by use of the log-rank (Mantel–Cox) test, and none of the comparisons between groups met our criteria to be considered significant (*P* ≤0.05). n.s. = not significant.

All children were initially enrolled in this pilot study based on *Cryptosporidium* spp. positivity using the QUIK CHEK test on day 0 stool samples. Therefore, all children in the NTZ plus probiotic arm (*n* = 26/26), NTZ plus placebo arm (*n* = 28/28), and control arm (*n* = 10/10) were 100% *Cryptosporidium* spp. positive using QUIK CHEK on day 0. By day 20, the infection rates as determined by QUIK CHEK test had significantly declined in all three groups (NTZ plus probiotic [23.1%; *n* = 6/26; *P* = 0.0001], NTZ plus placebo [25%; *n* = 7/28; *P* = 0.0001], and control [20%; *n* = 2/10; *P* = 0.0007]). No significant difference occurred between the groups (Figure 2A).

**Figure 3.**
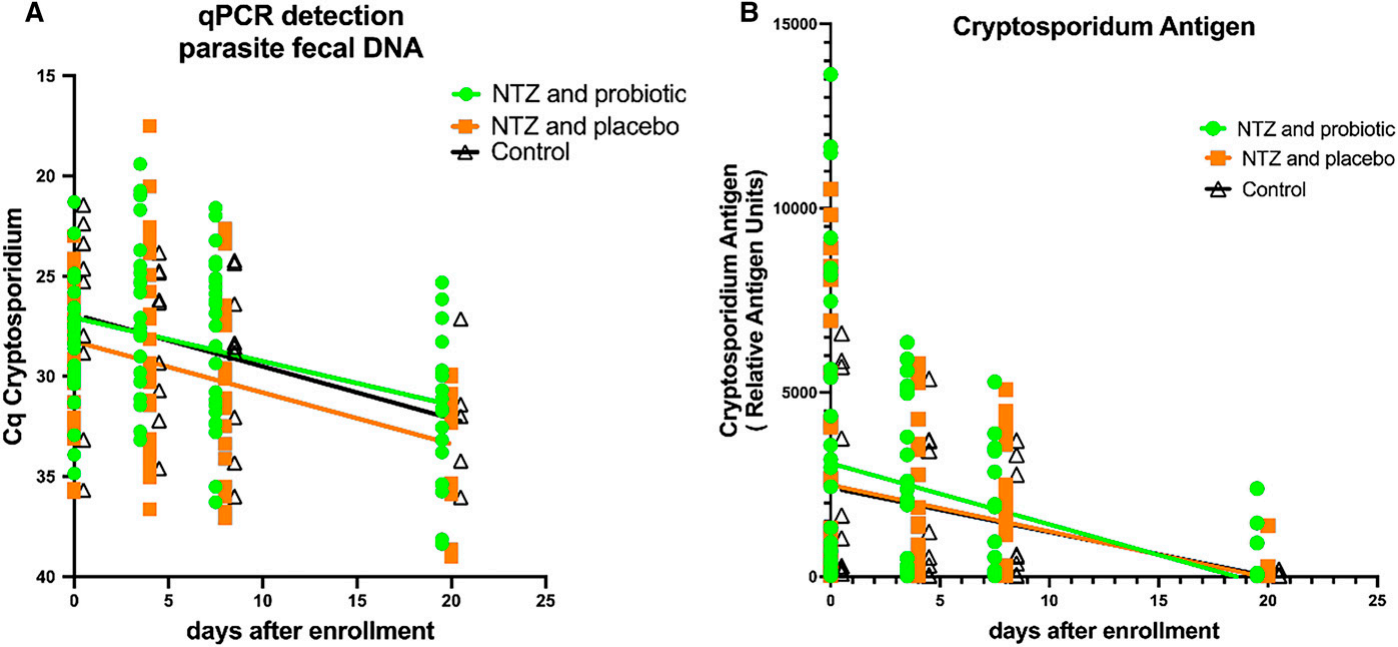
Relative change in parasite burden in the infected children over time. Impact of the different treatment regimens on parasite burden are measured by (**A**) diagnostic quantitative polymerase chain reaction (qPCR; the *y* axis indicates quantitative cycle [Cq]) and (**B**) *Cryptosporidium* antigen (the *y* axis indicates relative antigen levels). In both **A** and **B**, the *x* axes indicate the time in days since the child was enrolled; at each time point, each symbol represents the value obtained from an individual child, and the line represents the linear relationship between the parasite burden and the time infected in each group (simple linear regression): nitazoxanide (NTZ) plus probiotic (green symbols and lines), NTZ plus placebo (red symbols and lines), and controls (black symbols and lines). There were no significant differences in either the elevation or slope between any of the groups as assessed by linear regression.

The infection rates of *Cryptosporidium* spp. as measured by all three methods (QUIK CHEK, ELISA, and qPCR) over the course of 4 consecutive days are summarized in Table 1. At each time point, there were no significant differences in parasite burden as measured by antigen load (Figure 3A; Supplemental Figure 1) or parasite fecal DNA (Figure 3B) between the remaining infected children in each treatment group.

The ELISA assay identified fewer positive cases at day 0, but this did not change the outcome. The number of children positive by ELISA were as follows: NTZ plus probiotic arm, 84.6% (*n* = 22/26); NTZ plus placebo arm, 82.1% (*n* = 23/28); and control arm, 90% (*n* = 9/10). Infection rates in all three groups significantly declined on day 20 compared with day 0 (at the time of enrollment): NTZ plus probiotic, 11.5% (*n* = 3/22; *P* = 0.0001); NTZ plus placebo, 7.1% (*n* = 2/23; *P* = 0.0001); and control, 20% (*n* = 2/9; *P* = 0.005). No significant difference occurred between the groups (Figure 2B).

The diagnostic qPCR assay results (Figure 2C) agreed with the previous assay results, and no statistical difference was observed between the three groups. Generally, in this study, the qPCR analysis of fecal DNA had a greater sensitivity than antigen detection. However, at the time of enrolment (day 0), the children were 100% (*n* = 26/26), 96.4% (*n* = 27/28), and 90% (*n* = 9/10) positive by qPCR in the NTZ plus probiotic, NTZ plus placebo, and control arms, respectively. On day 20, infection rates decreased in the NTZ plus placebo (39.3%; *n* = 11/27; *P* ≤0.0001) and NTZ plus probiotic (61.5%; *n* = 16/26; *P* = 0.0007) groups, but the drop in the control arm (45%; *n* = 4/9) was not statistically significant because of the smaller group size.

### Child anthropometric measures were not appreciably altered by either NTZ or probiotic treatment.

The impact of NTZ with or without the probiotic on diarrhea could not be assessed in this study as only two symptomatic children were enrolled, and both were assigned to the NTZ plus placebo arm. Anthropometric measurements were collected at the time of study enrollment and during the follow-up visits at 90 and 180 days postenrollment. In all three groups, children’s weight, height, and MUAC were significantly increased compared with these values at enrollment. However, after correcting for child age, there was no improvement in HAZ, WAZ, and WHZ at 90 or 180 days in any of the treatment or control groups (Supplemental Figure 2). No “catch-up” growth was, therefore, observed in the NTZ with or without *Lactobacillus* or control groups at the 90- or 180-day follow-up visits.

## DISCUSSION

In our study, NTZ treatment either with or without probiotics did not have a discernible impact on any of the collected anthropometric measurements. Additionally, none of the three groups displayed any “catch-up” growth at the 90- or 180-day follow-up visits.

The primary limitation of this pilot study, like many others taking place in low- and middle-income countries, was the small sample size in each group, particularly in the control group. The effect of either NTZ or probiotic treatment on diarrheal cryptosporidiosis (Supplemental Tables 2 and 3) could not be established as only two cases of diarrheal *Cryptosporidium* spp. were enrolled in this study. In addition, the simple random sampling selection used in this study led to an uneven distribution of males and females in the different groups. The impact of this last limitation, however, is likely minimal because in a larger sample size involving children of this age group, no gender-based differences have been observed during cryptosporidiosis (W. A. Petri et al., unpublished data). In our study setting, it was also not possible to identify the exact day that infection began in the nondiarrheal subclinical *Cryptosporidium*-positive children. However, no significant differences were detected in the duration of parasite burden among the treatment groups.

The prevalence of *Cryptosporidium* spp. infection was, as expected, higher in diarrheal stool samples (9.1% of all diarrheal samples) than in nondiarrheal stool samples (3.1% of tested samples) which is consistent with the *Cryptosporidium* spp. infection prevalence of 3.3% found in asymptomatic stool samples in a prior study.^27^ In this trial, the number of *Cryptosporidium*-infected children as measured by immunodiagnostic methods decreased by day 20, indicating that this time frame is still a useful benchmark for the natural length of time for parasite clearance in this population. These results are in agreement with a prospective cohort study conducted in children in Bangladesh; index newborns shed *Cryptosporidium* for 19.9 days on average.^28^ As expected, the ELISA test results aligned with those of the QUIK CHEK test, which is reasonable because both are antigen-based assays. Significantly fewer infections were identified by QUIK CHEK on day 20 than determined by qPCR. In contrast to a study focused on diarrheal samples, the TechLab *Giardia/Cryptosporidium* spp. QUIK CHEK was less sensitive than qPCR in detecting ongoing parasite carriage during the infection time course of asymptomatic infections. This difference was not unexpected, however, because the qPCR assay is able to detect the fecal DNA from even small numbers of oocysts. In prior research, the sensitivity of the *Cryptosporidium* spp. qPCR was shown to be as low as 200 oocysts per gram of feces or 2 oocysts per polymerase chain reaction.^29^^,^^30^

Young children exhibit a significantly higher vulnerability to *Cryptosporidium* spp. infections compared with other age groups.^31^^,^^32^ Malnutrition is known to increase the risk of infection and may exacerbate the effects of illness, including *Cryptosporidium*. *Cryptosporidium* spp. infection in children also results in growth faltering.^33^^,^^34^ A single *Cryptosporidium* spp. infection during the first 2 years of life can disrupt a child’s physical development and lead to stunted growth by the age of 2 years.^5^ As mentioned previously, a limitation in our study was the small number of participants. Therefore, we only had the statistical power to detect major improvements in child health. It is also important to note that because we lacked a healthy control group without any infection, we cannot definitively conclude that *Cryptosporidium* spp. infection was the sole reason for the lack of nutritional status improvement. Our investigation did not find any evidence that *L. reuteri* DSM 17938 combined with NTZ treatment enhanced child growth in the context of HAZ, WAZ, and WHZ when compared with the NTZ arm.^35^ Similar to our investigation, the large randomized clinical trial (probiotics and prebiotics for severe acute malnutrition study) of Kerac et al.^36^ also did not find any positive effects of probiotic treatment on the health or nutritional status of malnourished children. In some animal studies, probiotics appear to be a useful therapy; however, in neonatal mice infected with *Cryptosporidium*, probiotic treatment was ineffective in improving the health,^37^ and the prolonged treatment with probiotics before *Cryptosporidium* infection in Holstein calves did not result in an improvement in animal health.^38^

## CONCLUSION

Although this pilot study does not rule out the possibility that NTZ may have subtle effects on health and parasite burden, this study demonstrated that NTZ therapy did not effectively treat cryptosporidiosis in children at risk of malnutrition and did not significantly decrease the duration of a *Cryptosporidium* spp. infection. Furthermore, the study findings suggest that *L. reuteri* DSM 17938 alone does not have a discernible effect on children with *Cryptosporidium* spp. infection. New approaches are needed to treat undernourished children most at risk from diarrheal cryptosporidiosis.

## Supplemental Materials

10.4269/ajtmh.23-0914 Supplemental Materials
