# Supplementary material for: Effect of Nitazoxanide and Probiotic Treatment on Bangladeshi Children with Cryptosporidiosis
Source: Am J Trop Med Hyg. 2025 Feb 11;112(4):733–9. doi: 10.4269/ajtmh.23-0914 (PMC11965742; doi:10.4269/ajtmh.23-0914)
Supplement: Supplemental Materials [file tpmd230914.SD1.pdf]

Supplementary Table 1

Comparison of infection outcomes between visit days measured by QUIK CHEK, ELISA and qPCR methods in fecal samples

Days of examination	QUIK CHEK assay		
	**NTZ+probiotic arm**	**NTZ+placebo arm**	**Control arm**
	**Positive (%)**	**Positive (%)**	**Positive (%)**
**Day-00**	26 (100)	28(100)	10 (100)
**Day-04**	21 (80.8)	19 (67.9)[¥]	9 (90)
**Day-08**	14 (53.8)[¥]	15 (53.6) [¥]	7 (70)
**Day-20**	6 (23.1) [¥,Ŧ,Ɨ]	7 (25.0) [¥,Ŧ,]	2 (20)[¥,Ŧ]
**ELISA assay**			
**Day-00**	22 (84.6)	23 (82.1)	9 (90)
**Day-04**	15 (57.7)	15 (53.6)[¥]	7 (70)
**Day-08**	13 (50.0)[¥]	13 (46.4)[¥]	6 (60)
**Day-20**	3 (11.5)[¥,Ŧ,Ɨ]	2 (7.1)[¥,Ŧ,Ɨ]	2 (20)[¥]
**qPCR assay**			
**Day-00**	26 (100)	27 (96.4)	9 (90)
**Day-04**	24 (92.3)	25 (89.3)	9 (90)
**Day-08**	23 (88.5)	23 (82.1)	9 (90)
**Day-20**	16 (61.5)[¥,Ŧ]	11 (39.3)[¥,Ŧ,Ɨ]	4 (40)

Data expressed as numbers (%). The chi-square test determined the P-value for the difference. ¥ = Day 4, 8 and 20 compared with day 0 at P <0.05; Ŧ= Day 8 and 20 compared with day 4 at P < 0.05; Ɨ = Day 20 compared with day 8 at P <0.05

Supplementary Table 2

Prevalence of cryptosporidium infection excluding diarrheal stools (n=26). Results show the *P*-values

Test Methods	Day 0 vs 4	Day 0 vs 8	Day 0 vs 20	Day 4 vs 8	Day 4 vs 20	Day 8 vs 20
**Quik chek**	0.0010	<0.0001	<0.0001	0.3965	0.0007	0.0479
**ELISA**	0.0197	0.0044	<0.0001	0.5780	0.0002	0.0010
**QPCR**	0.2980	0.0825	<0.0001	0.4421	<0.0001	0.0008

Supplementary Table 3

ELISA test of *Cryptosporidium* oocysts antigen excluding diarrheal stools (n=26). Results show the P-values

Test method	Day 0 vs 4	Day 0 vs 8	Day 0 vs 20	Day 4 vs 8	Day 4 vs 20	Day 8 vs 20
ELISA	0.0114	0.0014	< 0.0001	0.1996	0.0002	0.0011

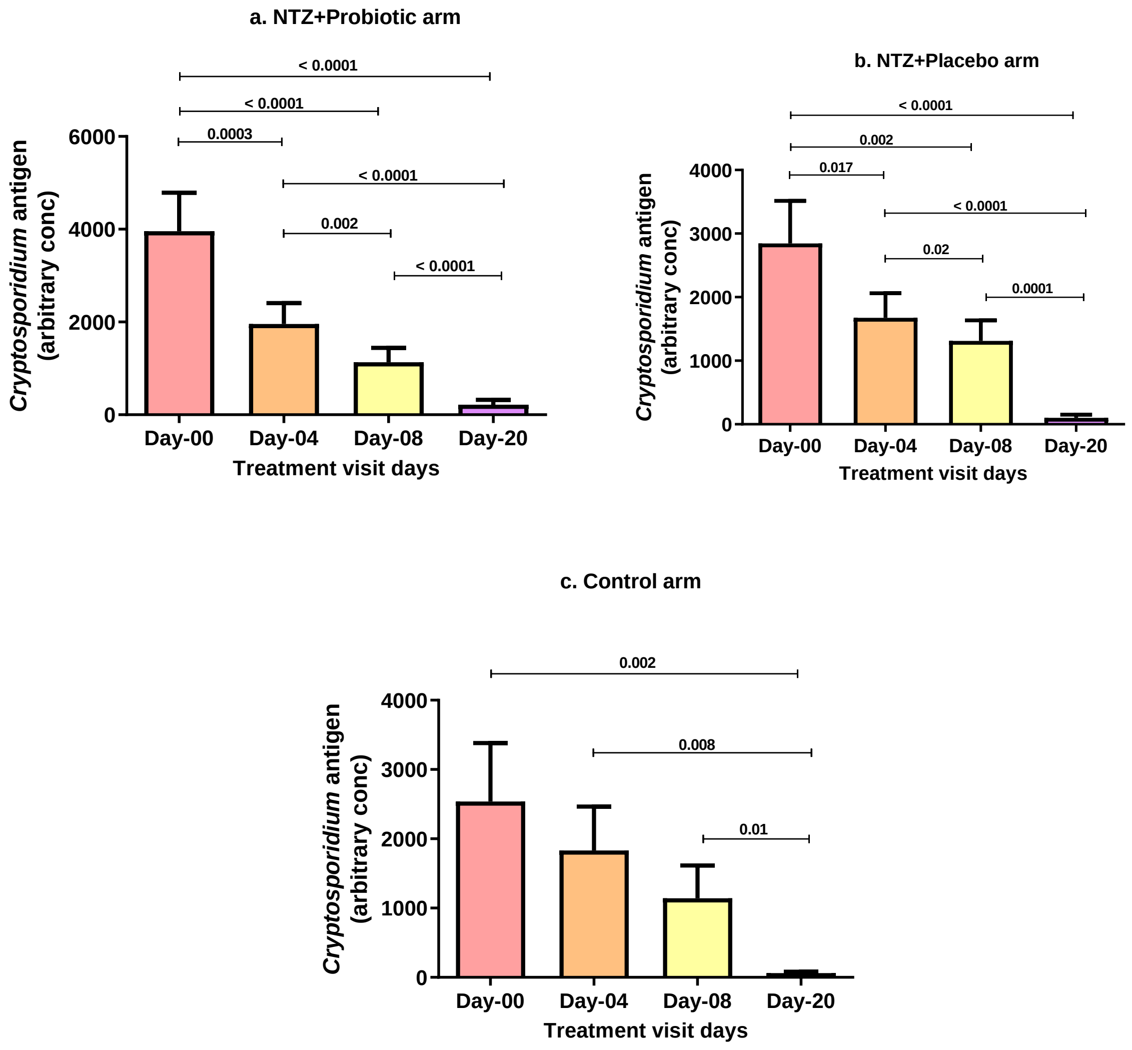

Supplemental Figure 1: ELISA test of *Cryptosporidium* oocysts antigen. Changes of optical density (OD) values at 450 nm of the ELISA plate readings of *Cryptosporidium spp.* oocysts antigen from fecal specimens. An arbitrary standard was used to quantify the relative antigen concentration of *Cryptosporidium* oocysts. The Kolmogorov-Smirnov normality test *was used to see the normality of* antigen *values.* The *P*-value for the difference was determined between days using the *Wilcoxon matched signed rank test.*

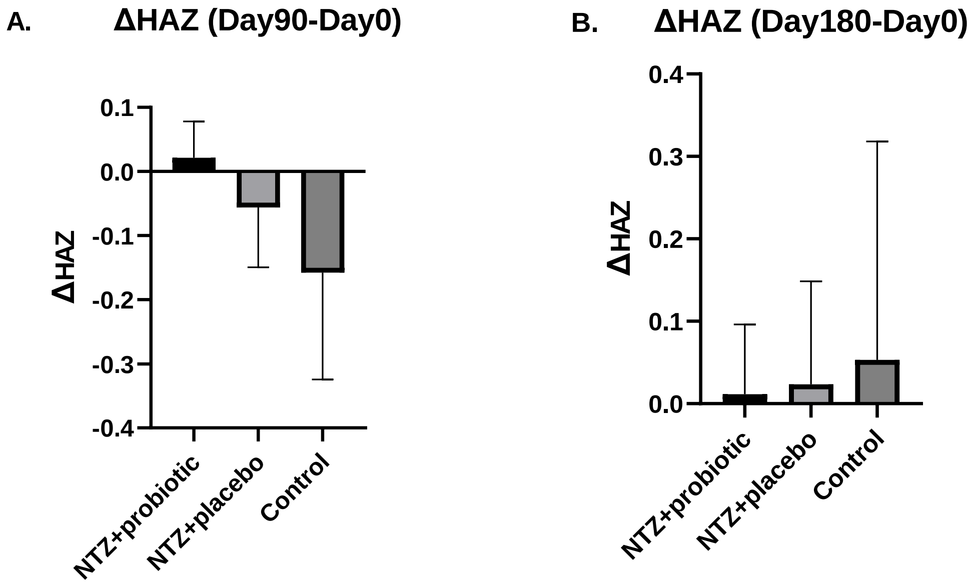

Supplemental Figure 2: Plots representing changes in delta HAZ in enrolled child in different groups (NTZ+probiotic, NTZ+placebo and control arms). Measurements taken at the time of enrollment, and at 90 days and 180 days follow up visits of the study supplementary figure 2(A) showing delta HAZ measured from day 90 to day 0. Supplementary figure 2(B) showing delta HAZ measured from day 180 to day 0. The *P*-value for the difference was determined between days using the unpaired t test.
